# Virtual Reality Simulation for Disaster Preparedness Training in Hospitals: Integrated Review

**DOI:** 10.2196/30600

**Published:** 2022-01-28

**Authors:** Younhyun Jung

**Affiliations:** 1 School of Computing Gachon University Seongnam-si Republic of Korea

**Keywords:** virtual reality, in-hospital disaster preparedness training, mass casualty incidents, hospitals

## Abstract

**Background:**

A critical component of disaster preparedness in hospitals is experiential education and training of health care professionals. A live drill is a well-established, effective training approach, but cost restraints and logistic constraints make clinical implementation challenging, and training opportunities with live drills may be severely limited. Virtual reality simulation (VRS) technology may offer a viable training alternative with its inherent features of reproducibility, just-in-time training, and repeatability.

**Objective:**

This integrated review examines the scientific evidence pertaining to the effectiveness of VRS and its practical usefulness in training health care professionals for in-hospital disaster preparedness.

**Methods:**

A well-known 4-stage methodology was used for the integrated review process. It consisted of problem identification, a literature search and inclusion criteria determination, 2-stage validation and analysis of searched studies, and presentation of findings. A search of diverse publication repositories was performed. They included Web of Science (WOS), PubMed (PMD), and Embase (EMB).

**Results:**

The integrated review process resulted in 12 studies being included. Principle findings identified 3 major capabilities of VRS: (1) to realistically simulate the clinical environment and medical practices related to different disaster scenarios, (2) to develop learning effects on increased confidence and enhanced knowledge acquisition, and (3) to enable cost-effective implementation of training programs.

**Conclusions:**

The findings from the integrated review suggested that VRS could be a competitive, cost-effective adjunct to existing training approaches. Although the findings demonstrated the applicability of VRS to different training scenarios, these do not entirely cover all disaster scenarios that could happen in hospitals. This integrated review expects that the recent advances of VR technologies can be 1 of the catalysts to enable the wider adoption of VRS training on challenging clinical scenarios that require sophisticated modeling and environment depiction.

## Introduction

Emergency preparedness in hospitals for disasters is unexpectedly required. Disasters could occur anywhere and at any time [1,2]. Disasters include not only accidents in hospitals (eg, an outbreak of fire) but also natural and human-made catastrophic events occurring outside of hospitals. In-hospital disaster preparedness must be performed fully on the spot, and health care professionals are the front line of disaster preparedness by controlling disaster situation and caring for victim patients. Human history has demonstrated the serious effects of poor disaster preparedness [3]. In the case of the aftereffect of Hurricane Katrina, the emergency evacuation at the Memorial Medical Center in New Orleans was unexpectedly conducted and poor evacuation management resulted in tremendous loss of life [4].

A fundamental component of effective in-hospital disaster preparedness is appropriate, repetitive experiential education and training of health care professionals with live disaster situations [5]. Disasters, however, are rarely encountered clinically, making it ideal for simulation. Live drills enable trainees to practice in a realistic simulation, where they can experience mock-up scenarios and learn sequences of actions and tasks to deal with the situations. Live drills are available when disaster simulations are set through the process of implementing everything realistically, including participants, infrastructures, and medical devices and tools. One of the live drill examples is the timely and effective evacuation practice of neonates during an outbreak of fire [6]. Here, fast exit routing by newborn intensive care unit workers while caring for mannequins of neonates had to be developed. Live drills have a great advantage in that they can take effect in a real disaster situation. Cost restraints and logistic constraints, however, make implementing simulations and scenarios difficult, and training opportunities with live drills may be severely limited [7,8].

Having lectures and practice with learning materials is another approach. Its advantages are that it is simple and does not require time and cost on a large scale. The simulations and instructions, however, may be fragmentary (eg, via verbal description or simple figures), and the learning outcomes may not be so effective in a real disaster situation [9].

Emerging evidence suggests that virtual reality simulation (VRS) is a viable alternative for workforce training in a variety of industry disciplines, with positive effects of increasing confidence and gaining necessary knowledge [10-13]. VR is a cutting-edge technology that can generate virtual environments of different training scenarios and combine with multisource data, stereoscopic vision, and intuitive interaction interfaces [14]. VR enables trainees to take part in virtual scenarios using avatars, in which they can simulate the sequences of actions and tasks they may make and reflect on the consequences of their choices [15,16]. Its realistic and interactive characteristics can greatly enhance trainees’ perception level and interest in learning [17]. The current generation of VR technologies is now equipped with head-mounted displays, where the wearers can obtain fully immersive experience of VRS [18]. There are various advantages of the use of VRS in education and training as follows: (1) repeatability, where training practices can be repetitive until goals are achieved [19]; (2) dynamic expansion and update of training scenarios due to technical advances to reconstruct a variety of training scenarios in a realistic manner [20,21]; and (3) just-in-time training, where training can be conducted anywhere and at any time if VR devices are available [22].

The purpose of this paper is to conduct an integrative review of peer-reviewed literature that applies VRS for in-hospital disaster preparedness training. The research problems this integrated review identified are follows: For use in in-hospital disaster preparedness, (1) *what kinds of VRS training programs have been presented?*; (2) *are there any studies that demonstrate the usefulness of the VRS training in a quantitatively or qualitatively manner, and does this usefulness analysis include technological feasibility, training effects, and cost saving of VRS?*; and (3) *are there any studies that identify the pros and cons of VRS training when compared to conventional alternatives (eg, live drills or material-based lectures)?*

This new integrated review builds upon and complements a prior integrated review publication by Miller et al [23]. In 2013, they analyzed peer-reviewed literature published during the period of 2005-2012, with findings that the efficacy of VRS for its use in disaster preparedness training was identified but such that the findings were difficult to be generalized due to the small volume of the literature. This new integrated review analyzed state-of-the-art literature published in the past 15 years, where there have been massive technological advances in VR engineering [17]. This motivated us to investigate the current progress in disaster preparedness training programs using VRS in terms of its wide adoption, practical usages, and efficiency. Compared to the prior publication [23], this new integrated review narrowed down the application domains and was only limited to in-hospital events and scenarios (eg, emergency departments) where health care professionals should equip patient triage and treatment skills for mass casualty incidents (MCIs) [24], which are critical and should be timely and carefully managed.

## Methods

### Study Design

This study followed the well-known methodology suggested by Whittemore et al [25] to conduct an integrative review. This review methodology consists of (1) problem identification, (2) a literature search and inclusion criteria definition, (3) validation of searched studies, and (4) presentation of findings. Two investigators—an assistant professor (author YJ) and a senior student of computer science—were involved in this study.

### Problem Identification

The investigators agreed on the purpose and boundaries of this study at the initial stage of the research meeting. It seems that health care professionals do not have enough opportunities for in-hospital disaster preparedness training [7,8]. The investigators also agreed on recent advances of VRS for its use in education and training in a variety of application industry domains [10-13]. The research problems mentioned in the Introduction section were then derived based on their agreement.

### Literature Search and Inclusion Criteria Definition

The literature search was performed by the investigators independently. They used 3 keywords: (1) “virtual reality,” which represents a key technology to be used for implementation of in-hospital disaster preparedness training programs; (2) “disaster” or “fire,” which represents catastrophic events to be simulated for training programs; and (2) “health care” and “hospital,” which represent major responders when the training programs are executed. The combination of these keywords can cover the volume of relevant literature for this integrated review. Note that the chosen keywords—“disaster” and “fire”—sufficiently covered different possible disaster types, where “disaster” represents natural and human-made catastrophic events (eg, earthquake and MCIs) and “fire” represents accidents the majority of the literature considers as catastrophic events occurring in hospitals.

A computerized search approach was used with 3 websites: Web of Science (WOS), PubMed (PMD), and Embase (EMB). WOS is an established publication repository for VR technologies (technology domains), and the other 2 are related to the medicine application domain. Such inclusion may decrease the possibility of missing relevant literature. The advanced search function of each publication repository was used as a search option as follows:

- The WOS search with its Web of Science Core Collection database and without any restrictions on document types
- The PMD search with all fields and without any other options or filters
- The EMB search with its default options

Inclusion criteria were as follows:

- Peer-reviewed empiric studies published from 2006 to 2020 and written in English
- All VRS training programs related to disaster preparedness
- Participants of training programs being all types of health care professionals
- Place of training programs anywhere in hospitals

### Validation of Searched Studies

The final studies to be included were determined through a 2-step validation procedure: (1) Abstracts of searched studies were initially reviewed to confirm whether they met the inclusion criteria, and (2) full papers of the abstract-filtered studies were printed and read in their entirety for inclusion. Many of the studies have contributed to the development of VRS training programs, and there was a lack of evaluating and analyzing their usefulness and efficacy in real practice scenarios. These were excluded since the purpose of this integrated review was to suggest the scientific evidence pertaining to the practical values of VRS training programs. The investigators independently performed the validation procedure. Discrepancies were resolved through active discussion among the investigators.

### Presentation of Findings

A matrix of the presentation for validated final studies was used to reach an integrated result. The matrix consisted of authors and dates of publication, study design, disaster type, participant details, contents, and outcomes. The investigators believed that this categorization would be useful to capture and promote the understanding of features of VRS disaster preparedness training programs and their usefulness and practical values. For quasi-experimental studies, it is important to identify differences and similarities, describe variables, and clarify intervention factors to make logical connections. All of these were carefully considered in this integrated review.

## Results

### Study Selection

There were 99 results (studies) from the literature search of the 3 publication repositories, as shown in Table 1. After study screening using the inclusion criteria and redundancy removal, 12 (12%) studies in total were included in this integrated review: 4 (24%) studies were included from 17 studies in WOS, 6 (26%) from 23 studies in PMD, and 2 (3%) from 59 studies in EMB.

**Table 1:** Numerical results of the literature search.

Search results	WOS^a^, n (%)	PMD^b^, n (%)	EMB^c^, n (%)	Total
Studies after initial literature search	17 (17.2)	23 (23.2)	59 (59.6)	99
Studies after screening using inclusion criteria	7 (32)	6 (27)	9 (41)	22
Final studies after redundancy removal	4 (33)	6 (50)	2 (17)	12

^a^WOS: Web of Science.

^b^PMD: PubMed.

^c^EMB: Embase.

### Study Review

The summarization for each of the 12 studies [6,24,26-35] is shown in Table 2 in ascending order of publication year. Here, there were 4 categories: authors and publication years, experiment, content, and outcomes. In the experiment category, data including the status of the randomized controlled trial (RCT) and disaster type and study design were described. In addition, the number of participants was provided, with a distribution of their role—either the experiment group (EG) with VRS training or the control group (CG) without VRS training—if a quasi-experimental study was conducted. Prior to experiments, both groups were taught through a conventional training approach (eg, lecture with learning materials) and an additional VRS training session was delivered only to the EG.

In Table 3, statistical characteristics of the 12 studies [6,24,26-35] are shown according to predefined categories.

**Table 2:** Summary of the 12 studies.

Author (year)	Experiment	Content	Outcome
Roy et al [26]	Case study Bioterrorism MCIs^a^ Physicians (number not specified) RCT^b^: N/A^c^ EG^d^: N/A CG^e^: N/A	In VRS^f^ sessions, participants were asked to conduct interviews with simulated patients and exams with virtualized resources (eg, medical images and heart sounds). Live drill simulations equivalent to the VRS sessions were also given for comparison. Qualitative and quantitative feedback was obtained to assess participants' performance, along with detailed conversational analysis. Software platform: SIMmersion	Analysis results suggested that VRS training can build skills, increase learning retention, improve trainee confidence, and change behaviors. VRS training requires greater initial investment but has lower marginal costs when compared to the live drill counterpart, and considerable return on investment.
Heinrichs et al [27]	Case study Bioterrorism MCIs 7 physicians and 6 nurses RCT: N/A EG: N/A CG: N/A	In VRS sessions, triage teams of participants assessed victims and sent them to appropriate treatment areas. The teams divided themselves into physician-nurse teams at each bedside and assessed and managed each virtual patient allocated. During the assessment, additional graphical information was given, and they performed intravenous infusions and administered blood and drugs. After completing the VRS sessions, an instructor facilitated a debriefing discussion. The participants were asked to conduct the same VRS sessions once more. After the second debriefing, they were asked to complete surveys and contribute to an open discussion. Most (9/13, 69%) of the participants were not gamers (69% never played VR systems), and most (8/13, 62%) had no prior training in responding to an MCI. Software platform: Online Interactive Virtual Environment (OLIVE)	Here, 8 (62%) of 13 participants reported that the VRS training changed their feelings and attitudes about working as members of an emergency department team. The ratings of the participants on the exit survey (5-point Likert-type scale: 1=low and 5=high) showed that they felt immersed (3.47) and thought that the VRS training increased their confidence in their ability to respond to MCIs (2.0 before training; 3.08 after training). Most also thought that the VRS training would be useful for learning teamwork skills and behaviors (3.77) as well as for learning the clinical skills necessary to treat MCI victims (3.15). Their comments also indicated they perceived the patient physiology models and virtual environment as realistic, although they would like the interface improved to allow them to perform a more rapid patient assessment.
Heinrichs et al [24]	Sequential study Bioterrorism MCIs 10 physicians (4 years of postgraduate experience) and 12 nurses (9.5 years of practice experience) RCT: N/A EG: N/A CG: N/A	Participants conducted VRS of 2 MCI sessions. For each session, they formed 2 teams—those assigned to a triage area and those assigned to an immediate treatment area—and began to act out their signed roles for assessing and treating victim patients. After the VRS sessions, the participants joined an instructor-led debriefing of their VRS performance and then filled out an exit questionnaire and contributed to a focus group discussion. Quantitative results were collected from a quiz that was administered at the beginning of the evaluation and an exit questionnaire that was completed at the end. The majority had never played VR games: the mean score on the frequency of play was 1.4 between “never” and “occasionally.” Approximately, two-thirds of the participants had previous triage training at some point prior to study enrollment. Software platform: OLIVE	Prior to the VRS training, only 4 (18%) of 22 participants were confident about managing MCIs. After the VRS training, 19 (86%) felt either “confident” or “very confident,” with 13 (59%) attributing this change to practicing in the VRS training. In addition, 21 (95%) reported that the session scenarios were useful for improving health care team skills training, and 18 (82%) believed that the sessions also were instructive in learning about clinical skill management of MCIs.
Pucher et al [28]	Sequential study Bomb terrorism MCIs 21 clinicians in 3 groups: 8 novices, 7 intermediates, and 6 experts RCT: N/A EG: N/A CG: N/A	In VRS, participants in each of the 3 groups were asked to form a team and required to perform clinical action to ensure appropriate place, transfer, and treatment for virtual patients. Participants were allowed to access additional information (eg, each patient's notes or vital signs). Technical skill performance of individual participants was collected on a 5-point Likert scale across a range of critical behaviors and tasks defined by a disaster planning expert panel. Nontechnical skill performance was scored based on the validated trauma nontechnical skills (T-NOTECHS). Scores were compared across groups. The participants filled a feedback and validity questionnaire, with statement responses on a 7-point Likert scale. Software platform: Unity	All 21 (100%) participants agreed that VRS would be an effective and realistic training tool for MCIs and that it was an enjoyable addition to their training and might help improve their own practice. The novice group committed more critical events than the expert group (11 novice vs 3 expert, P=.01), took longer to treat patients (560 seconds vs 399, P=.03), and resulted in poorer T-NOTECHS scores (14 vs 21.5, P=.003) and technical skill scores (2.29 vs 3.96, P=.001). Participants who previously underwent disaster response training thought that VRS has significant advantages over existing alternatives, but details of the advantages were not stated explicitly.
Ferra et al [6]	Quasi-experimental study Bioterrorism MCIs 106 nursing students RCT: yes EG: n=54 CG: n=52	Both EG and CG completed pretests of self-efficacy and cognitive learning. The EG conducted a VRS session where participants were required to practice sequential steps of decontamination skills with virtual tools (eg, donning personal protective equipment). After the VRS session, the EG completed posttests. Both EG and CG were then directed to a mannequin, on which they demonstrated decontamination while being evaluated and timed by an experienced observer. In total, 38 (35.8%) of 106 participants had previous disaster training prior to study enrollment. Software platform: not specified	The EG reported high levels of satisfaction with VRS as a training method. A significantly shorter amount of completion time from the EG was shown when compared to the CG (P=.01). The EG showed greater improvement in the self-efficacy score over the CG, although there was no significant difference (P=.17). No difference between the EG and CG was found in the cognitive knowledge score (P=.63).
Dorozhkin et al [29]	Sequential study Fire 49 physicians RCT: N/A EG: N/A CG: N/A	Participants were asked to complete an operating room fire training/prevention sequence given by a VRS session. They were then asked to answer a subjective preference questionnaire (5-point Likert-type scale) focused on the usefulness and fidelity of the VRS. Software platform: VEST^g^	Five questions focusing on VRS effectiveness and its usefulness in operating room fire safety training were rated above 4. The highest score (4.84) was given to the level of satisfaction of using VRS to learn the principles rather than just using textbooks, with the lowest score (2.95) associated with the quality of sensation of feeling the tools on the target and in the task space. A total of 33 (67%) of 49 participants chose VRS training over traditional approaches, such as a textbook or an animal model.
Dubovsky et al [30]	Sequential study Disaster not specified 10 nurses (25.3 years of practice experience) RCT: N/A EG: N/A CG: N/A	Participants mastered navigating VRS for code triage of virtual patients in an emergency department. They then participated in a testing scenario with code triage of a series of 6 virtual patients, which represented a range of severity and complexity. Participants decided which patient was seen next based on their assessment of priority. Attitudes toward the VRS and perceived workload in the VRS and on the job were assessed with an exit questionnaire and the NASA task load index. Only 1 (10%) of 10 participants had experience with VR games. Software platform: CliniSpace	Responses to the exit questionnaire indicated that the participants' attitudes toward the VRS were largely positive. Participants generally regarded the scenarios as realistic and perceived their work on the VRS task to be equivalent to their workload in a regular workday in all aspects except for physical exertion. The time to perform code triage corresponded to the time required in the emergency department, and virtual patients were appropriately prioritized according to severity.
Ferra et al [31]	Quasi-experimental study Disaster not specified 93 newborn intensive care unit health care works RCT: yes EG: at least 31 CG: at least 31	A longitudinal experiment was conducted to study both the EG and CG, with repeated measures taken at 0, 4, 8, and 12 months. Learning was measured using a cognitive assessment and self-efficacy questionnaire. The EG's qualitative experience was collected using a focus group. In addition, longitudinal performance was assessed with live evacuation drills before the study and 12 months after the study. In each period, the EG was asked to conduct VRS of emergency evacuation scenarios that augmented the materials developed by an established institution. The CG was asked to review the web-based lecture materials that deliver the same content as in the VRS. Software platform: not specified	The evaluation demonstrated mixed but overall positive results for the effect of VRS on newborn intensive care unit evacuation training. The EG and CG did not statistically differ based on the scores on cognitive assessment or perceived self-efficacy. The EG performance in the live evacuation drills, however, was statistically (P<.001) and clinically (effect size of 1.71) better than that of the CG. The EG showed slightly faster transfer of neonates, but this effect did not reach statistical significance.
Sankaranarayan et al [32]	Quasi-experimental study Fire 20 physicians RCT: not specified EG: 10 CG: 10	Both the EG and CG took a pretest that assessed the baseline knowledge in operating room fire and its prevention. The EG was asked to practice on a VRS session of a fire scenario within a week from the pretest. In the VRS session, the EG was asked to identify the elements of the fire triangle and the proper sequence of actions that needs to be taken if a virtual patient is on fire. A week after the posttest, both groups also participated in a live drill and simulated a mock-up fire scenario, while their performance was videotaped for assessment by 2 independent raters. Software platform: VEST	Median test scores for the CG increased from 5.5 to 9.00 (P=.01) and for the EG increased from 5.0 to 8.5 (P=.01). Both groups started at the same baseline (pretest, P=.53) and reached similar levels in cognitive knowledge (posttest, P=.85). When evaluated in the live drill, 7 (70%) of the EG participants were able to perform the correct sequence of steps in extinguishing the simulated fire, whereas only 2 (20%) of the CG participants were able to do that (P=.003).
Lovreglio et al [33]	Sequential study Earthquake 87 visitors and hospital staff RCT: yes EG: N/A CG: N/A	In total, 87 participants were randomly selected from 170 candidates. They were asked to practice a VRS session that was designed to generate training outcomes (ie, enhance participants' knowledge of how to behave in public and administrative areas of a hospital during and after an earthquake). The performance on the VRS session was assessed using a 6-point Liker scale questionnaire. Confirmatory factorial analysis was also run with the components forming the questionnaire. Software platform: Unity	The results from the questionnaire indicated that all its components received a positive score (eg, high rating score of 0.830 for the realism of the VRS environment). The component having the lowest score was realism of the nonplayer characters. The confirmatory factorial analysis result indicated that the realism of the virtual environment and the realism of earthquake simulation and damage play were the main contributing factors to the sense of presence available from the VRS. *U*-test results with the demographic information showed that there were no statistical differences related to the participant gender and type (staff vs visitors).
Rossler et al [34]	Quasi-experimental study Fire 20 prelicensure nursing students RCT: yes EG: 5 CG: 15	The EG completed a VRS training session designed for acquisition of knowledge of operating room fire safety. Both the EG and CG were then asked to complete a simulated fire live drill designed to assess the transfer of knowledge to practice settings. An investigator-developed fire safety evaluation test was administered in pretest (prior to the live drill)-posttest (after the live drill) format. Software platform: VEST	Both groups started at the same baseline in the acquisition of required knowledge (ie, the median score of 70 in the pretest). The EG showed a large increase (20 points) in gained knowledge compared with the CG (10 points), but there were no statistically significant findings for either group (between pre- and posttest).
Farra et al [35]	Quasi-experimental study Disaster not specified 91 newborn intensive care unit health care workers RCT: not specified EG: 34 CG: 57	A live drill was financially compared with the VRS counterpart. The costs of the live drill included exercise planning, exercise participants, exercise support, and exercise evaluation. Staff costs were based on the average hourly rate of representatives with a given title of those who were involved. The costs of the VRS included storyboard, consultants, training simulation development, travel from the development team, hardware supplies, and staff time for training participation. To have a meaningful comparison, the authors projected the cost of each alternative, assuming that all 334 staff members of the hospital were to undergo training once a year for 3 years. Software platform: Unity	The larger initial investment in the VRS can be spread across a large number of trainees and a longer time period with little additional cost, while each live drill requires additional costs that scale with the number of participants. Initially, the VRS was more expensive, with its cost of $327.78 per participant (the total cost of $106 951.14 per exercise) versus $229.79 (total cost $18 617.54) for the live drill. When development costs were extrapolated to repeated training over 3 years, however, the VRS training became less expensive, with a cost of $115.43 per participant, while the cost of live exercises remained fixed.

^a^MCI: mass casualty incident.

^b^RCT: randomized controlled trial.

^c^N/A: not applicable.

^d^EG: experiment group.

^e^CG: control group.

^f^VRS: virtual reality simulation.

^g^VEST: Virtual Electrosurgical Skill Trainer.

**Table 3:** Statistical characteristics of the 12 studies.

Variable	Category	Statistics, n (%)
**Publication year**		
	2006-2010	3 (25)
	2011-2015	2 (16)
	2016-2020	7 (59)
**Study design**		
	Case study design	2 (16)
	Sequential design	5 (42)
	Quasi-experimental design	5 (42)
**Disaster type**		
	Bioterrorism MCIs^a^	4 (34)
	Bomb terrorism MCIs	1 (8)
	Fire	3 (25)
	Earthquake	1 (8)
	Not specified	3 (25)
**Participants, n**		
	1-50	7 (59)
	51-100	3 (25)
	>100	1 (8)
	Not specified	1 (8)
**Experiment type**		
	RCT^b^	6 (50)
	No RCT	4 (34)
	Not specified	2 (16)
**Software platform**		
	Unity	3 (25)
	VEST^c^	3 (25)
	Online Interactive Virtual Environment (OLIVE)	2 (16)
	Others	2 (16)
	Not specified	2 (16)

^a^MCI: mass casualty incident.

^b^RCT: randomized controlled trial.

^c^VEST: Virtual Electrosurgical Skill Trainer.

## Discussion

### Principal Findings

All the 12 studies suggested positive outcomes of VRS from its use for in-hospital disaster preparedness training. Since the outcomes varied, the interpretation for the studies was multidimensional, focusing on 3 major themes: VRS training’s (1) realism, (2) learning effect, and (3) cost saving.

#### Realism of VRS Training

The definition of realism was how the health care participants felt that the training environment provided by VRS was like their actual clinical environment and medical practice. There were 4 (33%) of 12 studies [27,28,30,33] where the analysis and evaluation of VRS training programs with regard to realism was explicitly conducted. These studies [27,28,30,33] demonstrated the capabilities of VR technology in realistically simulating clinical scenarios and medical practices related to disasters. The confirmatory factorial analysis by Loverglio et al [33] suggested that such the replicability is the main contributing factor in the sense of realism. Here, the health care participants could interact with sequences of actions and tasks required to deal with the in-hospital disaster scenarios [27,28,30]. These findings may indicate that VR technology enables immersive training and learning in the clinical domain. This is consistently found in prior integrated reviews in medicine [23] as well as those in other nonmedical industry domains [12,13].

In a prior integrated review by Miller et al [23], triage and treatment training from human-made MCIs was only for application practices. This limited applicability was mitigated in the current integrated review by including new programs of building evacuation safety training from natural catastrophic events, such as earthquakes [33]. The technological advances in VRS during the past 15 years may be 1 of the contributing factors in the application to clinical scenarios that require sophisticated modeling and environment depiction.

There were complains about the VR interface from an early study by Heinrich et al [27] in 2008, but no such complaints were discovered in recent studies until 2020. This might imply that the technological maturity of VRS is at a level that includes consideration of extraneous variables, such as technological sophistication of the participants and ease of navigation.

#### Learning Effects of VRS Training

In total, 10 (83%) of 12 studies [6,24,26-32,34] (2 studies by Ferra et al [35] and Lovreglio et al [33]) evaluated VRS for its learning effects on in-hospital disaster preparedness training. Learning effects on increased confidence and enhanced knowledge acquisition were captured in all the 10 studies [6,24,26-32,34], and such learning effects were consistently found in previous integrated reviews in medicine [23] as well as other nonmedical industry domains [12].

In a prior integrated review by Miller et al [23], the learning effects were limited in triage and treatment knowledge and skills because all included studies focused on those practices. The larger variation of applied practices was found in our integrated review; it included not only triage practices but also training programs on fire prevention and safety, and emergency evacuation planning and execution. Through this new integrated review, it was found that the learning effects from the use of VRS can be generalized to a variation of medical training practices.

There were 4 (33%) quasi-experimental studies [6,31,32,34] where EG participants with VRS training were analyzed and directly compared with their CG counterparts without VRS training. These studies [6,31,32,34] consistently showed no significant difference between the 2 groups in posttest evaluation on self-efficacy or knowledge acquisition. The EG participants, however, showed statistical or clinical performance enhancement in gained knowledge and task completion efficiency when evaluated in final live drills (eg, performing the correct sequence of steps in extinguishing a simulated fire) [32]. This result suggested the practical usefulness of VRS and its learning effects on real disaster scenarios that health care professionals may encounter in the future.

One of the important learning effects is increased learning retention [36,37]. The prior integrated review by Miller et al [23], however, could not derive any findings on it due to a lack of studies. In this integrated review, there was 1 (8%) study [31] that took the learning retention effect into consideration. The study [31] conducted a longitudinal experiment with repeated measures taken during a year, and the results showed improved knowledge gaining as training progressed. This statement, however, is somewhat difficult to be generalized due to the insufficient volume of longitudinal experimental studies. This insufficiency may be, in part, attributed to the fact that inputs and efforts, including participants, facilities, and program management, are much greater and have to be maintained for a longer period when compared to 1-shot studies.

#### Cost Saving of VRS Training

Cost analysis of the training program implementation and operation is 1 of the important factors training bodies have to consider, but it was not part of the previous integrated review by Miller et al [23]. In this integrated review, 2 (16%) of 12 relevant studies [26,35] suggested consistent findings for the cost advantage in implementing and operating VRS training programs over the live drill alternative. The larger initial investment from VRS training was undeniable, but it could be scalable and compensated for its longer-term use with the large number of participants. A comparative numerical study by Farra et al [35] found that the initial additional cost from VRS training was $97.99 when compared to the live drill alternative, and this initial investment provided a benefit of $114.65 after 3 years of program operation. Farra et al [35] attributed this to the repeatability feature of VRS anywhere and anytime, enabling just-in-time training.

#### Other Aspects of VRS Training

This integrated review of 12 studies [6,24,26-35] showed the applicability of VRS to different training scenarios of in-hospital disaster preparedness, including triage and transport of victim patients, emergency treatment, fire extinguishing, and building evacuation. These, however, do not entirely cover all disaster scenarios that could happen in hospitals. Investigating VRS with other challenging clinical scenarios that require sophisticated environment depiction and complicated training tasks (eg, patient protection and evacuation in scenarios of water entering a hospital due to floods) is an interesting future direction for effective in-hospital disaster management. The recent maturity of VR technologies in modeling and rendering and the development tools would be able to address the complexity of different clinical scenarios.

The number of VRS studies on in-hospital disaster preparedness training programs is still limited. Compared to the previous integrated review by Miller et al [23], the number increased from 10 to 12. Considering that Miller et al [23] collected all the VRS studies, including training scenarios occurring outside hospitals and in-hospital events, the increase in VRS studies on in-hospital training programs is noticeable. In addition, it was observed that the number of studies published during the past 5 years (from 2016 to 2020) was greater than that published in the older but longer period between 2006 and 2015 (ie, 7 [29-35] vs 5 studies [6,24,26-28]).

The scale of participants is 1 of the important variables to draw conclusive findings from studies. There were 4 (33%) of 12 studies [6,31,33,35] that considered the scale of participants by conducting VRS training programs with more than 50 participants. This increased from 2 in the previous integrated review by Miller et al [23] and was double. In addition, all 4 studies [6,31,33,35] were conducted in the recent 5 years. These observations suggest that future studies would put more emphasis on this important variable and the potential outcomes can be more conclusive.

It was observed that 7 (58%) of 12 studies [29-35] since the past 5 years used consistent development platforms, such as the Virtual Electrosurgical Skill Trainer (VEST) and Unity, for the clinical implementation of VRS training programs. The use of established development platforms enables rapid implementation, easy scenario replication, and, therefore, increased feasibility in conducting VRS-based clinical training studies. This observation can somewhat explain the increasing trends in the number of VRS studies and studies with large-scale participants.

It was also observed that the details of evaluation processes were not fully described. There were only 3 (25%) of 12 studies [24,28,30] that explicitly stated the skill levels and experiences of health care participants, and 1 (8%) study [26] even lacked details of the participant number. Demographic details can be 1 of the influential variables to allow in-depth interpretation of outcomes from studies (eg, how learning effects work for different levels of expertise, as shown in the study by Pucher et al [28]). Similarly, qualitative analysis in the participants’ experience was described using Liker-type surveys, postexperience interviews, and focus group open discussions, where there were little details on the reliability and validity of coding of qualitative data from interviews or focus groups. Such a lack was consistently found in the previous integrated review by Miller et al [23]. This integrated review, therefore, suggests that process details need to be sufficiently described in future studies, which will facilitate future investigators to draw deep insights into the value of VRS.

It was noted that the baseline performance comparisons of different studies highly varied: 3 (25%) of 12 studies compared VRS training programs among participants where some had previous disaster preparedness training experiences prior to study enrollment; 6 (50%) studies [6,26,27,30-32,34,35] compared VRS training effects against the experiences of live drills; and the remaining 3 (25%) studies [28,29,33] did not explicitly state the comparison approach. This may suggest that some efforts on developing the standard of VRS training program comparison protocols are meaningful, and this will allow for objective and quantitative comparison among different studies and draw general insights that are applicable to different disaster training programs.

### Conclusion

The findings from this integrated review suggest that VRS could be a viable, cost-effective approach for health care professional training in in-hospital disaster preparedness. The reproducibility, just-in-time training, and repeatability features of VRS, along with its low cost of clinical implementation, suggest that VRS potentially represents a competitive adjunct to existing training approaches.

As VR continues to evolve in all technological aspects, it is anticipated that studies using VRS can become more vitalized in the clinical domain, while addressing currently unsolved issues. As an example of issues, studies with a massive number of participants with sophisticated assessment tools can be performed with more detailed and rigorous interventions and measurement of long-term retention.

## Abbreviations

CG: control group
EG: experiment group
EMB: Embase
MCI: mass casualty incident
PMD: PubMed
RCT: randomized controlled trial
VEST: Virtual Electrosurgical Skill Trainer
VR: virtual reality
VRS: virtual reality simulation
WOS: Web of Science
